# Antiviral Efficacy of Metal and Metal Oxide Nanoparticles against the Porcine Reproductive and Respiratory Syndrome Virus

**DOI:** 10.3390/nano11082120

**Published:** 2021-08-20

**Authors:** Simon P. Graham, Yuen-Ki Cheong, Summer Furniss, Emma Nixon, Joseph A. Smith, Xiuyi Yang, Rieke Fruengel, Sabha Hussain, Monika A. Tchorzewska, Roberto M. La Ragione, Guogang Ren

**Affiliations:** 1 School of Veterinary Medicine, University of Surrey, Guildford GU2 7AL, UK; summerfurniss@hotmail.co.uk (S.F.); enixon@ncsu.edu (E.N.); j.smith9617@gmail.com (J.A.S.); rieke.fruengel@gmail.com (R.F.); sabha96h@gmail.com (S.H.); monica_tchorzewska@yahoo.co.uk (M.A.T.); r.laragione@surrey.ac.uk (R.M.L.R.); 2 The Pirbright Institute, Woking GU24 0NF, UK; 3 School of Physics, Engineering and Computer Sciences, University of Hertfordshire, Hatfield AL10 9AB, UK; y.cheong2@herts.ac.uk (Y.-K.C.); x.yang5@herts.ac.uk (X.Y.)

**Keywords:** porcine reproductive and respiratory syndrome virus, antiviral nanoparticle, viricidal efficacy, *staphylococcus aureus*

## Abstract

Porcine reproductive and respiratory syndrome viruses (PRRSV) are responsible for one of the most economically important diseases affecting the global pig industry. On-farm high-efficiency particulate air (HEPA) filtration systems can effectively reduce airborne transmission of PRRSV and the incidence of PRRS, but they are costly, and their adoption is limited. Therefore, there is a need for low-cost alternatives, such as antimicrobial filters impregnated with antiviral nanoparticles (AVNP). During the past 10 years, tailored intermetallic/multi-elemental AVNP compositions have demonstrated effective performance against human viruses. In this study, a panel of five AVNP was evaluated for viricidal activity against PRRSV. Three AVNP materials: AVNP2, copper nanoparticles (CuNP), and copper oxide nanoparticles (CuONP), were shown to exert a significant reduction (>99.99%) in virus titers at 1.0% (*w*/*v*) concentration. Among the three, CuNP was the most effective at lower concentrations. Further experiments revealed that AVNP generated significant reductions in viral titers within just 1.5 min. For an optimal reduction in viral titers, direct contact between viruses and AVNP was required. This was further explained by the inert nature of these AVNP, where only negligible leaching concentrations of Ag/Cu ions (0.06–4.06 ppm) were detected in AVNP supernatants. Real-time dynamic light scatting (DLS) and transmission electron microscopic (TEM) analyses suggested that the mono-dispersive hydrodynamic behavior of AVNPs may have enhanced their antiviral activity against PRRSV. Collectively, these data support the further evaluation of these AVNP as candidate nanoparticles for incorporation into antimicrobial air-filtration systems to reduce transmission of PRRSV and other airborne pathogens.

## 1. Introduction

Porcine reproductive and respiratory syndrome (PRRS) is caused by two species of PRRS virus (PRRSV-1 and -2; *Betaarterivirus suid 1 and 2*, respectively, order *Nidovirales*, family *Arteriviridae*) [1]. PRRSV are enveloped, single-stranded, positive-sense RNA viruses with a tropism for porcine cells of monocyte/macrophage lineage [2]. PRRSV initially infects alveolar macrophages [3], and replication in the lungs is important for aerosol spread through respiratory secretions [4]. PRRSV are highly infectious, with only 10 virions required to experimentally infect pigs [5]. PRRS continues to be one of the most economically significant swine diseases globally. A recent estimate of the annual cost due to PRRS in the USA alone was US $664 million [6]. Reproductive disease incurs a large portion of the cost due to abortions and/or the birth of weak piglets. Although respiratory symptoms associated with PRRSV may not cause mortality, they affect growth and sales weight. Both species of PRRSV are rapidly evolving, and highly pathogenic strains emerge sporadically [7,8,9,10], which poses a major ongoing threat to the swine industry worldwide.

Currently, the control of PRRS is best achieved through a combination of vaccination and biosecurity measures, such as segregation and disinfection. However, the aerosol transmission of PRRSV has been demonstrated experimentally. It is becoming more widely accepted as a common route of transmission, including for farms to become re-infected following the elimination of PRRSV from a herd [11,12,13,14]. Although air filtration systems do not eliminate the risk of new virus introductions, they can limit the spread. US pig farms not equipped with an air filtration system were eight times more likely to experience a PRRS outbreak than farms that used air filtration [15]. It was also estimated that sow farms that utilized air filtration could produce 5927 more pigs per year than an unfiltered farm, using a model of a hypothetical 3000 sow farm [16]. A four-year study comparing the efficacy of commercial air filters, including mechanical fiberglass, antimicrobial and electrostatic mechanical fiberglass filters, showed that all prevented the spread of PRRSV from infected to susceptible pig populations [17]. Air filtration systems appear to be a highly effective PRRS control tool, but they may be expensive to install and operate, needing to be replaced often [18]. This is not optimal, especially for smaller farms, and an air-filtration system with antimicrobial filter pads doped or impregnated with antiviral nanoparticles (AVNP) could provide a cost-effective approach [19,20].

AVNP and antimicrobial nanomaterials have been shown to possess a range of biomedical functionalities [21,22,23,24]. AVNP with intermetallic/multi-elemental compositions have shown to actively counteract a range of bacterial pathogens [25,26], H5N1 and H1N1 influenza A viruses and the severe acute respiratory syndrome coronavirus (SARS-CoV) [27]. In addition, these nanoparticles can act at low concentrations in suspensions under the environmental water threshold level (<100 ppm) [28,29]. AVNP represent prime candidates for impregnation into fibers [19] and the development of air filtration systems to aid in the control of PRRSV. As a critical first step, this study aimed to investigate the antiviral efficacy of AVNP against PRRSV, utilizing liquid suspensions. From an initial screening of five AVNP, three were selected for further study. A potent and rapid antiviral action of a novel intermetallic-carbon complex nanoparticle (AVNP2) and copper nanoparticles (CuNP) were characterized. We also demonstrated a broader antimicrobial effect of this AVNP against *Staphylococcus aureus*, a Gram-positive bacterium prevalent in pig populations globally and of public health importance.

## 2. Materials and Methods

### 2.1. Antiviral Nanoparticles (AVNP)

Antiviral nanoparticle second-generation (AVNP2), copper oxide nanoparticles (CuONP), zinc oxide nanoparticles (ZnONP), and tungsten carbide nanoparticles (WCNP) were produced using a Tesima™ thermal plasma technology [30,31,32] from QinetiQ Nanomaterials Ltd., Farnborough, UK. AVNP2, developed by the UK Antiviral Nanoparticles Consortium, is composed of an sp^2^ hybridized graphitic carbon and W-Ag-Cu metallic complex, manufactured for an antiviral purpose and customized for bioengineering applications [27]. Both AVNP2 and CuONP have spherical shapes. The average particle size range of AVNP2 and CuONP were between 10–20 nm and 10–70 nm, respectively. WCNP are hexagonal in shape with an average hydrodynamic diameter of 250 nm. Elemental copper nanoparticles (CuNP) were purchased from CF Nanotechnology (Suzhou, China). All the raw AVNP materials used in this study were further analyzed by scanning electron microscopy (SEM) prior to the preparations of AVNP suspensions for in vitro evaluation. Representative SEM micrographs of WCNP, AVNP2, CuNP, CuONP, and ZnONP are shown in Supplementary Figures S1–S5, respectively. The engineered WCNP showed no antiviral/antimicrobial effect against both virus (PRRSV) and bacteria (*S. aureus*) at the concentrations tested. They were treated as an additional negative control in this study. 

Antiviral/bacterial assays required all AVNP to be in suspension form by dispersing into saline, PBS, or water at an initial concentration of 1.0% (*w*/*v*). All AVNP suspensions were prepared by sonication to disperse each powder into an aqueous medium with an on/off pulse program at 53% working power applied to each sonication process. The sonication device was a 750 W high-frequency liquid processor (Sonics & Materials, Newtown, CT, USA). All materials were sonicated for 2–3 min until well-dispersed suspensions were formed.

### 2.2. PRRSV Propagation and Titration

The PRRSV-1 subtype 1 strain Olot/91 was kindly provided by Dr. Jean-Pierre Frossard, Animal and Plant Health Agency, Addlestone, UK. The virus was propagated in MARC-145 cells cultured in Dulbecco’s modified Eagle’s medium (DMEM) supplemented with sodium pyruvate, GlutaMAX, 100 U/mL penicillin, 100 µg/mL streptomycin, and 10% fetal bovine serum (all Thermo Fisher Scientific, Loughborough, UK) (cDMEM). PRRSV infectious titers were determined by 10-fold serial dilution of viruses, added to 96 well flat bottom tissue culture plates containing 5 × 10^3^ MARC-145 cells/well. The plates were incubated at 37 °C with 5% CO_2_ for 3 days. Immunoperoxidase staining was performed to determine the number of infected wells and allow calculation of the median 50% tissue culture infective dose (TCID_50_) [33]. PRRSV was detected using nucleocapsid protein-specific monoclonal antibody 1AC7 (Ingenasa, Madrid, Spain), followed by the addition of rabbit anti-mouse immunoglobulin HRP conjugate (Thermo Fisher Scientific, Abingdon, UK) and staining visualized by the addition of DAB substrate (Sigma Aldrich, Poole, UK).

### 2.3. Assessment of Antiviral Activity of AVNPs against PRRSV In Vitro

Freshly prepared suspensions of AVNPs in PBS serially diluted 10-fold in cDMEM were added to an equal volume of the stock suspension of PRRSV and incubated at room temperature for 1 h with vortexing every 5 min. The same treatment of the virus with PBS was included as a negative control. PRRSV/AVNP mixtures were then centrifuged for 4 min at 3000× *g* at room temperature to pellet AVNP. A log_10_ dilution series of the supernatant was created, which was then added to triplicate wells in a 96 well plate seeded with MARC-145 cells and infectious titers determined as described above. An experiment was additionally performed by varying the incubation time of the virus with AVNPs, from 60 to 30, 15, 7.5, 5, 2.5, and 1.5 min. A final experiment utilized the supernatants from suspensions of AVNPs (prepared by centrifugation after 1 h incubation at room temperature) and incubated with PRRSV to assess antiviral activity, as described above.

### 2.4. Assessment of AVNP Cytotoxicity

AVNP suspensions were serially diluted in cDMEM and added to MARC-145 cells in 96 well white-walled plates (Fisher Scientific, Loughborough, UK). After 24 h incubation, cytotoxicity was assessed using the CellTiter-Glo^®^ Luminescent Cell Viability Assay (Promega, Southampton, UK). Following the addition of substrate, luminescence was measured using a CLARIOstar multilabel plate reader (BMG LabTech, Aylesbury, UK).

### 2.5. Inductively Coupled Plasma Atomic Emission Spectroscopy Sample Preparation and Elemental Analyses

All labware was acid washed overnight with 4 M nitric acid prepared from 70% nitric acid (Fisher Scientific, Loughborough, UK) and rinsed thoroughly with deionized water before use. For ion release measurements (AVNP leaching analysis), 20 mL of dispersed nanoparticle suspensions in (1 mg/mL, 1000 ppm) were left standing at room temperature for 24 h when the clear settlement of nanoparticles was observed. Then, 1 mL of the supernatants from each sample were collected and filtered using 0.22 µm sterile syringe filters (Millipore Millex^TM^, Thermo Fisher Scientific, Abingdon, UK) prior to analysis using a Varian 710 Inductive Coupled Plasma-Optical Emission Axial Spectrophotometer (ICP-OES; Varian, Frankfurt, Germany) equipped with a SeaSpray Nebulizer. Emission intensities of three different wavelengths from both Ag (λ = 241.318, 328.068, 338.289 nm) and Cu (λ = 231.598, 324.754, 327.395 nm) were detected, technical and software triplicates were obtained from each AVNP sample. Ag and Cu content in supernatants were fully quantified against external calibration curves standardized using TraceCert^®^ analytical grade standards for ICP (Sigma-Aldrich GmbH, Schnelldorf, Switzerland) with a weighted regression. The relative Ag/Cu concentrations (in ppm) were then calculated using the formula y = mx + c. Fully digested raw powders of AVNP that showed significant antiviral activity (AVNP2, CuNP, and CuONP) were subjected to full metal trace elemental analysis using ICP-OES (Supplementary Figures S12–S15).

### 2.6. Dynamic Light Scattering (Zeta Potential & Nanoparticle Tracking Analysis)

Zeta potentials were measured using a Nano ZS Zetasizer (Malvern Panalytical, Malvern, UK) at 25 °C using a standard operation protocol for the AVNP samples. Aqueous suspensions of each nanoparticle stock (0.1% *w*/*v*) were dispersed in water as previously described. Selected concentrations (10, 50, and 100 ppm) of each AVNP sample were freshly prepared prior to loading into a plastic cuvette (Malvern Panalytical). Each nanoparticle analyte was measured in triplicate. Nanoparticle tracking analysis (NTA) was performed using an LM10 NanoSight Instrument (Malvern Panalytical) equipped with a green laser source (λ = 532 nm) and a CMOS camera. Each nanoparticle dispersant was directly loaded into a sample guard; individual particles were detected using a static method. Brownian motion of the nanoparticles in aqueous suspension was recorded in real-time at 21 °C. The conditions for each analysis were optimized by adjusting the camera shutter, gain, and laser power. Each tracking analysis was captured using in-built software (NTA 3.2 Dev Builds 3.2.16) and was collected for 30 s in triplicate.

### 2.7. Surface Morphology Observation Using Scanning and Transmission Electron Microscopy

The surface morphologies of nanomaterials were analyzed using SEM and transmission electron microscopy (TEM). Prior to the preparation of AVNP suspensions, the morphology of nanomaterials was observed using SEM (JEOL JCM-5700, Tokyo, Japan). All SEM samples were dried under vacuum and coated with 2–3 nm of gold using an Emitech SC7620 Sputter (Quorum Technologies, Laughton, UK) prior to the image acquisitions. All images were collected and processed using an in-built software within the instrument. TEM images of CuNP, AVNP2, CuONP, and ZnONP samples were acquired using a JEOL JEM-1400 instrument (Tokyo, Japan) operating at 200 kV (Supplementary Figures S6–S11). For this study, nanopowders were sonicated first in 1 mL of ethanol, and then two drops of the suspension were placed on 200 mesh copper grids with Formvar and carbon supports (Agar Scientific Ltd., Essex, UK). Each sample’s excess solvent was removed under reduced pressure (10 mmHg) prior to TEM image acquisition.

### 2.8. Assessment of the Minimum Bactericidal Concentration of AVNP

A *Staphylococcus aureus* isolate (SA2; NCTC 178582), a methicillin-resistant (RC1; NCTC 12493), and a methicillin-susceptible *S. aureus* strain (SC1; NCTC 12981) were obtained from the University of Surrey Strain Collection (Guildford, UK) and cultured aerobically on brain heart infusion (BHI) agar (Sigma Aldrich, Poole, UK) at 37 °C for 24 h. AVNP suspensions were serially diluted 2-fold in Mueller-Hinton broth (MHB; Sigma) (1–0.007%) and added to 96 well plates containing an equal volume of MHB and 1 × 10^5^ colony forming units (cfu) of *S. aureus*. Plates were incubated at 37 °C, aerobically in a shaking incubator at 150 rpm for 24 h. Following incubation, a 20 μL aliquot of each sample was plated out onto MH agar and incubated at 37 °C, aerobically for 24 h before assessment of bacterial growth. The minimum bactericidal concentration (MBC) was determined as the lowest broth dilution of AVNP, which prevented *S. aureus* growth on the agar plate. To assess the effects of AVNP concentration on *S. aureus* growth over time, suspensions of AVNP (diluted from 0.03%, 0.015% and 0.007% (*w*/*v*) to avoid impairments with spectrophotometry readings) and *S. aureus* were incubated as described above, and optical density (600 nm) measured after 0, 8, 16 and 24 h using a Spark multimode plate reader (Tecan, Männedorf, Germany).

### 2.9. Statistical Methods

One- or two-way ANOVA’s with Dunnett’s or Tukey’s multiple comparison tests with a 95% confidence interval were performed in GraphPad Prism v7.01 (GraphPad, La Jolla, CA, USA) to statistically analyze the data (viral titer data were log_10_ transformed prior to statistical analysis).

## 3. Results

### 3.1. Evaluation of the Antiviral Activity of AVNP against PRRSV

**Figure 1:**
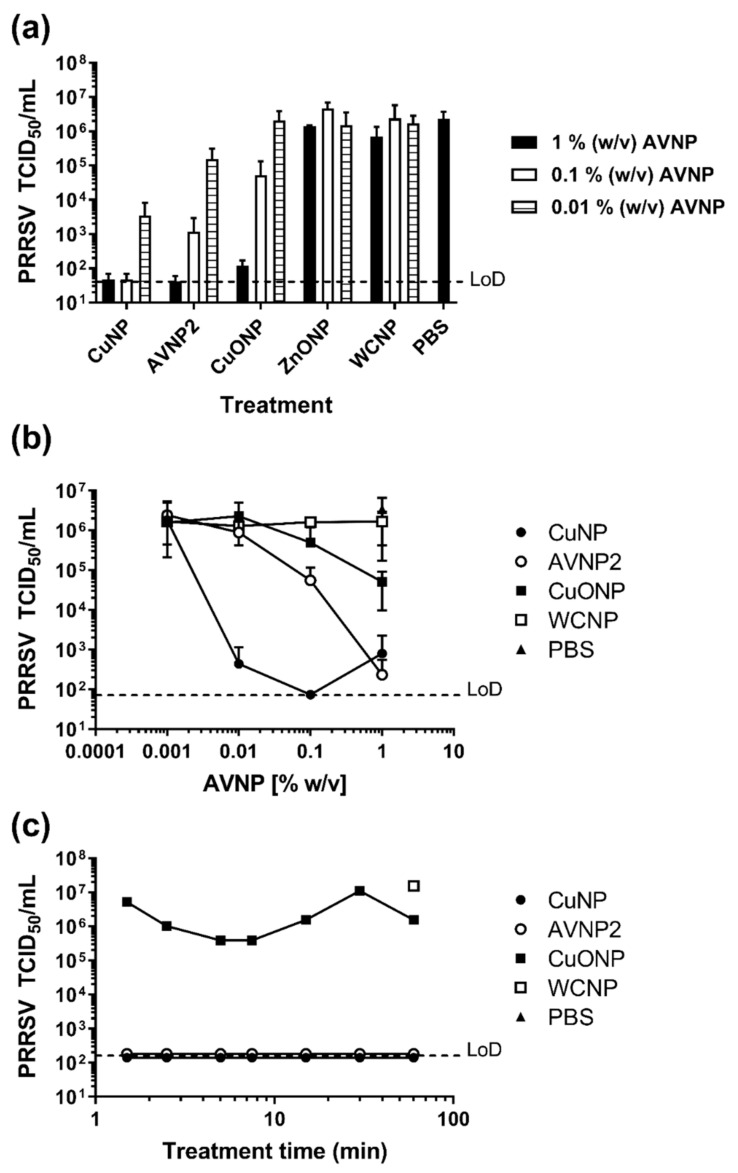
Assessment of the antiviral activity of AVNP against PRRSV. (**a**) PRRSV-1 Olot/91 was exposed to AVNP suspensions at 1.0, 0.1 and 0.01% (*w*/*v*) concentrations for 1 h at room temperature. PRRSV treated with PBS served as a negative control. After removal of the AVNP, infectious virus titers (TCID_50_) were obtained. Mean data ± SD from two-three independent assays is shown. (**b**) The three most effective AVNP (CuNP, AVNP2, and CuONP) were further titrated, alongside WCNP and PBS as negative controls. Mean data ± SD from four independent assays is shown. (**c**) The effect of incubation time on the antiviral activity of CuNP, AVNP2, and CuONP at 1% (*w*/*v*) concentration was assessed at 7 different times ranging from 1.5–60 min. Single technical replicates are shown for each time point. Horizontal dashed lines indicate the limit-of-detection (LoD) of the assays.

Independent assays were performed measuring the activity of a panel of AVNP (CuNP, AVNP2, CuONP, ZnONP, and WCNP) against PRRSV. The effects of pre-treatment with AVNP suspensions at 1, 0.1 and 0.01% (*w*/*v*) for 1 h at room temperature on the infectious titers of PRRSV-1 Olot/91 strain were assessed (Figure 1a). The virus was undetectable following exposure to CuNP at both 1.0 and 0.1% (*w*/*v*) concentrations, whereas the mean titer of virus recovered following treatment with 0.01% (*w*/*v*) CuNP was 10^3.54^ TCID_50_/_mL_. Compared to the mean titer of the control PBS treatment (10^6.36^ TCID_50_/mL), this equated to a >48,658-fold reduction in infectious titer for both 1.0 and 0.1% (*w*/*v*) (*p* < 0.001) and a 659-fold reduction for 0.01% (*w*/*v*) (*p* < 0.01). Treatment with AVNP2 was also highly effective with a clear dose-dependent effect. Mean virus titers following treatment with 1.0, 0.1, and 0.01% (*w*/*v*) AVNP2 were 10^1.61^, 10^3.07^, and 10^5.19^ TCID_50_/_mL_ (representing a 55,199, 1929 [both *p* < 0.001] and 15 [*p* < 0.05] fold reductions, respectively). Exposure of virus to CuONP led to a less pronounced dose dependent reduction in viral titer; with 1.0, 0.1, and 0.01% (*w*/*v*) reducing titers to 10^2.08^ TCID_50_/_mL_ (18,714-fold reduction; *p* < 0.001) 10^4.72^ TCID_50_/_mL_ (48-fold reduction; *p* < 0.05) and 10^6.31^ TCID_50_/_mL_ (2-fold reduction), respectively. Treatment of PRRSV with ZnONP and WCNP did not produce a significant reduction in titer when compared to the PBS control at any concentration tested.

Further assays were performed to confirm the efficacy of CuNP, AVNP2, and CuONP against PRRSV and suspensions were further diluted (1.0, 0.1, 0.01, and 0.001% (*w*/*v*) to titrate out the antiviral activity Figure 1b. Compared to the WCNP and PBS control treatments, 1.0% (*w*/*v*) suspension of CuNP, AVNP2 and CuONP all caused a significant reduction in PRRSV titers (*p* < 0.05). However, the difference between AVNP2 and CuNP at 1.0% (*w*/*v*) was not significant, both AVNP2 and CuNP produced a greater reduction in viral titer compared to CuONP (*p* < 0.0001). At a concentration of 0.1% (*w*/*v*), only CuNP and AVNP2 significantly reduced viral titers (*p* < 0.05), with CuNP producing the lowest viral titer of the two (CuNP: 10^1.86^ TCID_50_/_mL_ and AVNP2: 10^4.28^ TCID_50_/_mL_; *p* < 0.0001). The CuONP treatment condition at 0.1% (*w*/*v*) did not differ from the control treatments. Compared to the control and both AVNP2 (10^5.35^ TCID_50_/_mL_) and CuONP (10^6.05^ TCID_50_/_mL_), CuNP produced a significantly lower viral titer of 10^2.2^ TCID_50_/_mL_ at 0.01% (*w*/*v*) (*p* < 0.0001). All particles at 0.001% (*w*/*v*) were ineffective at reducing viral titer (*p* > 0.05).

To assess how quickly AVNP can exhibit their effect against PRRSV, 1% (*w*/*v*) suspensions were evaluated with seven different incubation times, as shown in Figure 1c. The two most effective particles, CuNP and AVNP2 exhibited the same antiviral effect at all incubation times tested (1.5, 2.5, 5, 7.5, 15, 30, and 60 min), both nanoparticles reducing titers to below the assay’s limit of detection (<10^1.55^ TCID_50_/_mL_). CuONP produced titers ranging between 10^3.38^–10^4.72^ TCID^50^/^mL^, which were comparable between incubation periods.

### 3.2. Investigation of the Possible Interaction between AVNP and PRRSV

**Figure 2:**
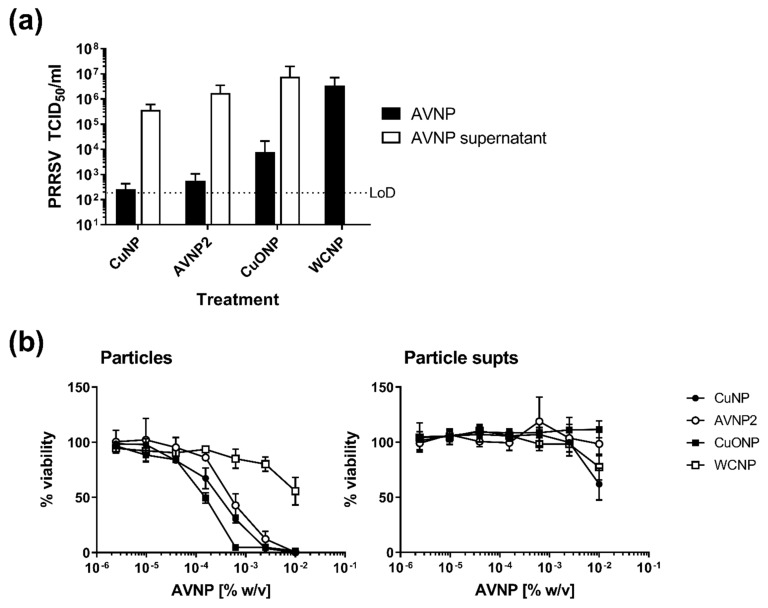
Assessment of the requirement for direct contact to mediate the antiviral activity of selected AVNP. (**a**) Supernatants from 1.0% (*w*/*v*) CuNP, AVNP2, and CuONP suspensions (incubated aerobically for 1 h at room temperature) were prepared by centrifugation and compared alongside 1.0% (*w*/*v*) AVNP suspensions for their ability to inactivate PRRSV-1. WCNP served as the negative control. AVNP suspensions/supernatants were incubated with the virus for 1 h at room temperature before centrifugation and determination of infectious virus titers (TCID_50_). Mean data ± SD from three technical replicates is shown. A horizontal dashed line indicates the limit-of-detection (LoD) of the assay. (**b**) The cytotoxicity of AVNP and suspension supernatants were additionally assessed by addition to MARC-145 cell cultures and assessment of cell viability after 24 h. Data are presented as the mean % viability ± SD (calculated using the control PBS treatment condition as 100%) for technical triplicate treatments.

We hypothesized that virus destruction occurred due to ions released from AVNP in suspensions. Thus, an experiment was conducted to compare the antiviral activity of AVNP supernatants (containing proposed ions) against the particles (Figure 2a). Incubation of PRRSV with each of the three AVNP (CuNP, AVNP2, and CuONP) at 1.0% (*w*/*v*) concentration resulted in significantly reduced viral titers (*p* < 0.01) compared to incubation of virus with the comparable AVNP supernatants or the WCNP and PBS controls. CuNP and AVNP2 supernatants slightly reduced viral titers, but this was not significantly different from the negative controls (*p* > 0.05). Thus, it appears that direct contact is necessary to mediate the anti-PRRSV activity of the selected AVNP.

To assess whether direct contact with AVNP would exert cytotoxic effects, MARC-145 cells were cultured with serial dilutions of AVNP (CuNP, AVNP2, CuONP, and WCNP) or their supernatants and toxicity assessed, as shown in Figure 2b. Percentage viability for each treatment was calculated using values from the PBS control wells as 100%. There was little variation in the dose-dependent toxicity between CuNP, AVNP2, and CuONP treatments, with an average of 6.3% viability at 0.01% (*w*/*v*) concentration (10 ppm) to an average of 81.7% viability at the lowest concentration (0.00000244%; 0.024 ppm). When comparing these values with that of the negative control AVNP sample (WCNP supernatant), higher concentrations (0.01%, 0.0025%, and 0.000625%) of CuNP, AVNP2, and Cu0NP were significantly toxic (*p* < 0.0001). At 0.000156% (*w*/*v*) (1.56 ppm) and 0.0000391% (*w*/*v*) (0.391 ppm) concentrations, only CuNP and AVNP2 remained statistically different from the control (*p* < 0.01). However, at the two lowest concentrations tested, all three AVNP were not significantly different from the PBS or WCNP controls (*p* > 0.05). The supernatants from all the AVNP exhibited little cytotoxic effect, the only significant data arose from AVNP2 and CuONP supernatants tested at 0.01% (*w*/*v*) (100 ppm), deeming these preparations cytotoxic compared to the control (*p* < 0.05).

**Figure 3:**
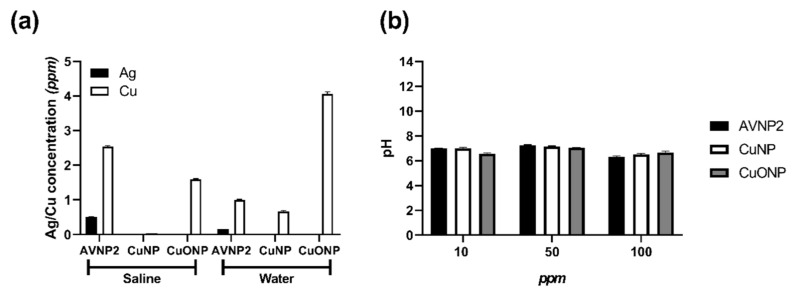
Investigation of the leaching and ionic properties of AVNP2, CuNPs, and CuONPs. (**a**) Ag and Cu present in AVNPs supernatants were fully quantified against standardized calibration curves with a weighted regression by ICP-OES. (**b**) The pH level of AVNP suspensions were measured at different concentrations (10, 50, and 100 ppm).

To understand the insufficient antiviral effects produced by the AVNP supernatants Figure 2, the presence of saturated ions and, hence, the metallic trace ions (Cu and Ag) leached from bare AVNPs, were examined by ICP-OES using a fully quantitative method. As shown in Figure 3a, the leaching properties of CuNPs, AVNP2, and CuONPs at concentrations of 0.1% (*w*/*v*) (1000 ppm) were assessed by measuring the metal content released into two different types of aqueous supernatants (saline and water). In saline, AVNP2 released the highest concentration of Cu (2.54 ppm), followed by CuONP (1.59 ppm) and negligible release from CuNP (0.06 ppm). In contrast, Cu release in water was the greatest from CuONPs (4.06 ppm), followed by AVNP2 (1 ppm) and CuNPs (0.66 ppm). As expected, Ag release was only detected from the AVNP2 samples with a higher concentration in the saline suspensions than water (0.5 ppm vs. 0.16 ppm, respectively). To rule out the physiochemical involvement of the corresponding antiviral activity, pH values of the three effective AVNPs (AVNP2, CuNPs, and CuONPs) suspensions were measured at various concentrations (10, 50, and 100 ppm), and all were found to be near the pH neutral (pH 7.0 ± 0.7) Figure 3b.

**Figure 4:**
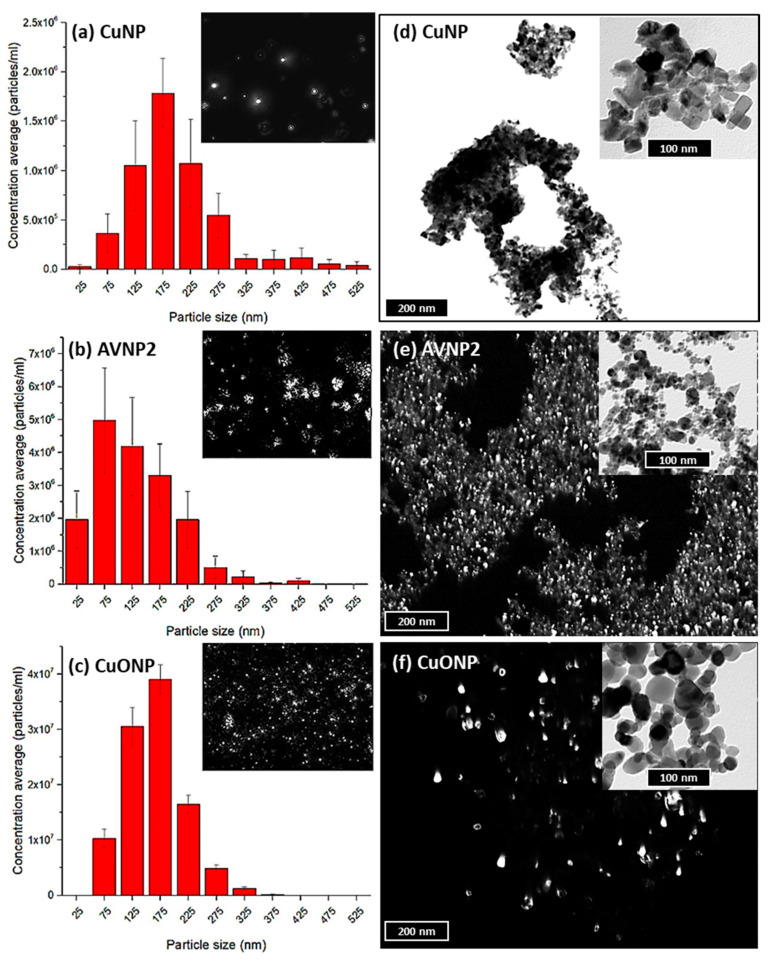
Investigation of the hydrodynamic behavior of AVNP in aqueous suspensions (left column) and morphology of AVNP at solid states (right column). (**a**–**c**) shows the hydrodynamic sizes, distributions, and concentrations of CuNP, AVNP2, and CuONP suspensions at 500 ppm, obtained from by DLS study using a nanoparticle tracking analyzer (NTA). The histograms illustrate the numbers of particles per mL (±SD) plotted against particle size distribution ranging from 0–525 nm, whilst the corresponding inserts show images of the hydrodynamic dispersive behavior of individual AVNP in an aqueous medium captured in real-time. (**d**) shows bright-field TEM images of bare CuNP with some clear rectangular/cubic crystal shapes. (**e**,**f**) shows the dark field TEM images of both AVNP2 and CuONP, which demonstrated the presence of crystallinity within the bare samples. The inserts in (**e**,**f**) showed the corresponding bright-field image of AVNP2 and CuONP at a higher magnification. Enlarged bright and dark field TEM images of AVNP are shown in Supplementary Figures S5–S9.

As the antiviral effect appeared to involve direct particle interactions, further morphological analyses were performed using TEM and DLS to understand how the physical properties of raw AVNP in their solid states and their hydrodynamic behavior in aqueous states may attribute to their interaction with PRRSV. Nanoparticle tracking analysis (NTA) was used to capture the dynamic light scattering of AVNPs (CuNP, AVNP2 and CuONP) suspended in water. As shown in Figure 4a–c, the resultant DLS from AVNPs suspensions were recorded and quantified to give numerical information of the nanoparticles’ hydrodynamic sizes and concentrations (particles/mL). The average hydrodynamic sizes of all three AVNP were found to be similar, mainly ranging from 25–275 nm. However, larger particles could be seen in the CuNP suspension ranging between 325–525 nm. Although the NTA real-time images suggested both AVNP2 (Figure 4b insert) and CuONP (Figure 4c insert) suspended well under aqueous conditions, they both appeared to be rather aggregated compared to the aqueous CuNP sample Figure 4a insert.

TEM images of the dry CuNP, AVNP2, and CuONP nanopowders provided details of the shapes and sizes of the tested AVNP. They revealed the presence of crystallinity in the AVNP and CuONP samples. These can be seen in Figure 4e,f, where bright spots demonstrated crystalline strongly diffracted in the dark field mode during TEM image acquisitions. These observations were in line with their manufacturing methods in which the AVNP2 and CuONP were engineered through a complete vaporization top-down process. The process uses high-temperature plasma to vaporize all microscale particles and condenses the metal gases in the processing downstream to form nanoparticles involving a recrystallization formation. As no crystalline structure was found in CuNP, images in Figure 4d only show the TEM results in bright field mode. The crystallinities of AVNP2 and CuONP have been discussed previously [30,34].

The surface charge (zeta potential in mV) of each effective AVNP at a concentration of 50 ppm was analyzed using a Zeta-sizer (another DLS technique). It was found that both CuNP and AVNP2 had negative surface charges at −9.27 (±2.68) and −20.63 (±1.9) mV, respectively, whereas CuONP showed a positive surface charge of +8.4 (±0.66) mV.

### 3.3. Assessment of the Antimicrobial Activity of Selected AVNP against S. aureus

**Figure 5:**
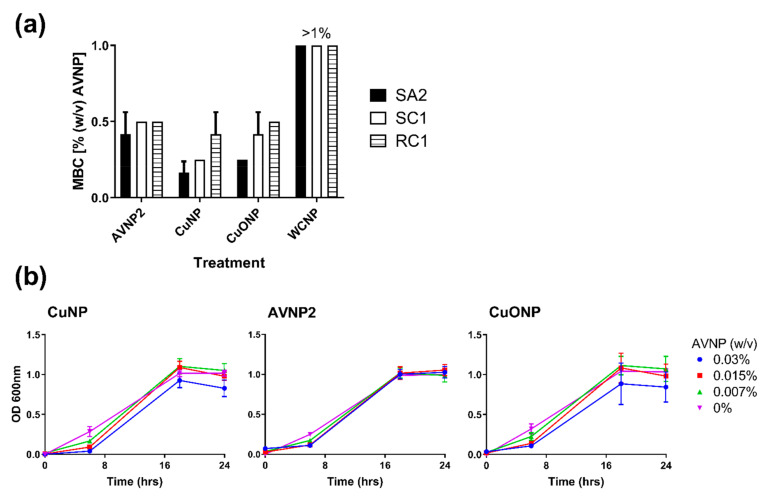
Assessment of the antimicrobial activity of selected AVNP against *S. aureus*. (**a**) The minimum bactericidal concentration (MBC) (% *w*/*v*) of AVNP2, CuNP, CuONP, and WCNP was determined against three *S. aureus* strains (SA2, SC1, RC1). Mean data ± SD from three independent assays is shown. (**b**) The inhibition of *S. aureus* growth (strain RC1) in the presence of 0.03% (*w*/*v*), 0.015% (*w*/*v*) and 0.007% (*w*/*v*) suspensions was assessed over time. Mean data ± SD for the three *S. aureus* strains collected in two independent experiments are shown.

As a final element to this study, the utility of the selected AVNP was assessed further by evaluating their activity against the bacterium *S. aureus*. Methicillin-resistant *S. aureus* (MRSA) occurs widely in pig populations globally, and while colonization alone does not cause disease in pigs, it is of great public health importance [35,36]. AVNP2, CuNP, and CuONP all showed bactericidal activity against the three *S. aureus* strains tested, whilst WCNP showed no bactericidal effect at any of the concentrations tested. For comparison to AVNP2, CuNP, and CuONP, the minimum bactericidal concentration (MBC) of WCNP was given a nominal value of 1, as shown in Figure 5a. CuNP showed the lowest MBC, displaying complete inhibition of growth at 0.17%, 0.25%, and 0.42% (*w*/*v*) for the three test *S. aureus* strains, which were significant compared to WCNP (*p* < 0.0001). AVNP2 showed significant growth inhibition against the three strains with an MBC of ~0.5% (*w*/*v*) (*p* < 0.001). CuONP also exhibited clear antimicrobial activity against all three strains, MBC of 0.25%, 0.42%, and 0.5% (*w*/*v*) (*p* < 0.001). In contrast to the antiviral activities, the differences in MBC between AVNP2, CuNP, and CuONP were not statistically significant. AVNP inhibition of bacterial growth was assessed by estimating bacterial concentration by measuring optical density (OD) at 600 nm Figure 5b. AVNP suspensions were tested at 0.03% (300 ppm), 0.015% (150 ppm), and 0.007% (*w*/*v*) (70 ppm) since higher particle densities impaired OD measurements. CuNP and CuONP suspensions both significantly reduced bacterial growth (*p* < 0.001 and *p* < 0.05, respectively), whereas AVNP2 did not. CuNP at 0.03% (*w*/*v*) significantly reduced growth as measured at 8 and 24 h (*p* < 0.01) and at 0.07% (*w*/*v*) only reduced growth at 8 h (*p* < 0.01). Other AVNP suspensions did not show significant reductions at individual time points (*p* > 0.05).

## 4. Discussion

This study has shown that CuNP and AVNP2 possess potent antiviral activity against PRRSV. Results consistently showed a >99.9% reduction in virus titer, which is commonly considered the required level of reduction for an effective disinfectant. Nevertheless, CuNP particles were more efficient at lower concentrations than AVNP2, in line with previous literature finding copper-based nanoparticles to be a particularly effective antimicrobial [30]. CuNP also showed the most effective antimicrobial activity against *S. aureus*. Our previous study showed that whilst CuNP showed a good antimicrobial effect against Gram-positive *S. aureus* and Gram-negative *Pseudomonas aeruginosa*, a more pronounced effect was seen with multi-elemental nanoparticles, such as AVNP2 [26]. But this did not translate into antiviral activity against PRRSV. On investigating the contact time required for PRRSV destruction, it was found that AVNP2 and CuNP can have their full effect after just 1.5 min of incubation at room temperature. However, further research is required to test even shorter incubation times to clarify the minimum contact time required for virucidal activity. This would be an important factor for consideration if they were to be applied in filtration systems. 

Past research has suggested that viral or bacterial destruction could result from ions released from metallic nanoparticles [28,29,30]. However, results from the assay directly comparing the antiviral activity of particles and their supernatants showed that AVNP2, CuNP, and CuONP particles were far more effective at destroying PRRSV than their own supernatants. ICP-OES analysis showed these AVNP to be rather inert; only an average of 0.13% of active ions was found to have saturated into the corresponding aqueous supernatants. Thus, the lack of active ion presence in the AVNP supernatants would explain the supernatant experiments’ low effects for both antiviral and cytotoxic activity. Moreover, the low ionic concentrations of the CuNP supernatant (0.06−0.66 ppm) also suggested that strong antiviral effect observed was not related to the ionic interactions between CuNP and PRRSV. PRRSV is inactivated by acidic conditions [37], but AVNP suspensions presented a near-neutral pH, ruling this out as an explanation for the antiviral activity.

As the observed antiviral effects of all three AVNP (CuNP, AVNP2, and CuONP) involved direct ‘particle-virus’ interactions, the initial mode of contact would involve the surface of the AVNP and the PRRSV virion. Therefore, we next analyzed the AVNP themselves. The DLS analysis showed evidence of mono-dispersibility of CuNP in an aqueous state that may have increased chances of NP exposure. It provided a high surface contact level with PRRSV, which led to an enhanced antiviral effect. It is worth noting that once CuNP was dispersed into an aqueous medium, and it was expected to slowly oxidize into cuprous oxide (Cu_2_O), which is a highly reactive species with cytotoxic and antiviral activity [38,39]. Zeta potential measurements found that both CuNP and AVNP2 had negative surface charges, whereas the less effective CuONP showed a positive surface charge. Hence, the negatively charged CuNP and AVNP2 may be statically attracted to the PRRSV virion, leading to more effective virion destruction. A similar explanation had been reported elsewhere [34].

In summary, this study has demonstrated the potent ability of AVNP2, CuONP, and CuNP in the liquid phase to inactivate PRRSV and, to a lesser extent, *S. aureus*. Evaluation of AVNP physicochemical properties provided data that may partly explain the toxicity of these particles to viruses, bacteria, and mammalian cells. These data enabled the selection of AVNP as candidate nanoparticles for further evaluation, which will involve the preparation of hybrid AVNP embedded polymer fibers [19]. Retention of efficacy at solid/gas interfaces would enable the development of novel antimicrobial air-filtration systems to reduce transmission of PRRSV and other airborne pathogens.

## Data Availability

The data presented in this study are available on request from the corresponding authors.

## Supplementary Materials

The following are available online at https://www.mdpi.com/article/10.3390/nano11082120/s1, Figure S1. SEM image of WCNP.; Figure S2. SEM image of AVNP2.; Figure S3. SEM image of CuNP.; Figure S4. SEM image of CuONP.; Figure S5. SEM image of ZnONP.; Figure S6. TEM image of CuNP (bright field).; Figure S7. TEM image of CuONP (bright field).; Figure S8. TEM image of CuONP (dark field).; Figure S9. TEM image of AVNP2 (bright field).; Figure S10. TEM image of AVNP2 (dark field).; Figure S11. TEM image of ZnONP (bright field).; Figure S12. Elemental analysis of blank nitric acid (HNO_3_) sample, where each optical emission spectrum (OES) shows the detected signal of the corresponding traced metal. As can be seen, the blank nitric acid was found to contain Zn, Si, Ca, Mg. It is worth noting that these signals also appeared in the subsequent results obtained from all AVNP samples (Figures S12–S14), as nitric acid was used for the digestion process, these observed signals therefore were regarded as blank signals.; Figure S13. Metal trace elemental analysis of fully digested CuNP using ICP-OES. Apart from a trace of Iron (Fe), Copper was the only element detected in the fully digested CuNP sample.; Figure S14. Metal trace elemental analysis of fully digested CuONP using ICP-OES. The manufacturing method, morphological and antimicrobial studies of CuONP have been previously published by Ren et al., 2009 (DOI: 10.1016/j.ijantimicag.2008.12.004).; Figure S15. Metal trace elemental analysis of fully digested AVNP2 using ICP-OES. The composition of AVNP2 has been previously studied, further structual characterization was published by Cheong et al., 2017 (doi:10.3390/nano7070152).
