# Supplementary material for: Antiviral Efficacy of Metal and Metal Oxide Nanoparticles against the Porcine Reproductive and Respiratory Syndrome Virus
Source: Nanomaterials (Basel). 2021 Aug 20;11(8):2120. doi: 10.3390/nano11082120 (PMC8398903; doi:10.3390/nano11082120)
Supplement: Supplementary file 1 [file nanomaterials-11-02120-s001.zip › nanomaterials-1277710-supplementary.pdf]

Article

Antiviral Efficacy of Metal and Metal Oxide Nanoparticles Against the Porcine Reproductive and Respiratory Syndrome Virus

Simon P. Graham [1,2,*], Yuen-Ki Cheong [3], Summer Furniss [1], Emma Nixon [1], Joseph A. Smith [1], Xiuyi Yang [3], Rieke Fruengel [1], Sabha Hussain [1], Monika A. Tchorzewska [1], Roberto M. La Ragione [1] and Guogang Ren [3,*]

[1] School of Veterinary Medicine, University of Surrey, Guildford GU2 7AL, UK; summerfurniss@hotmail.co.uk (S.F.); enixon@ncsu.edu (E.N.); j.smith9617@gmail.com (J.A.S.); rieke.fruengel@gmail.com (R.F.); sabha96h@gmail.com (S.H.); monica_tchorzewska@yahoo.co.uk (M.A.T.); r.laragione@surrey.ac.uk (R.M.L.R.)
[2] The Pirbright Institute, Woking GU24 0NF, UK
[3] School of Physics, Engineering and Computer Sciences, University of Hertfordshire, Hatfield AL10 9AB, UK; y.cheong2@herts.ac.uk (Y.-K.C.); x.yang5@herts.ac.uk (X.Y.)
* Correspondence: simon.graham@pirbright.ac.uk (S.P.G.); g.g.ren@herts.ac.uk (G.R.); Tel.: +44-(0)1483-231-478 (S.P.G.); +44-(0)7940-767-589 (G.R.)

Citation: Graham, S.P.; Cheong, Y.-K.; Furniss, S.; Nixon, E.; Smith, J.A.; Yang, X.; Fruengel, R.; Hussain, S.; Tchorzewska, M.A.; La Ragione, R.M.; et al. Antiviral Efficacy of Metal and Metal Oxide Nanoparticles Against the Porcine Reproductive and Respiratory Syndrome Virus. *Nanomaterials* **2021**, *11*, x. https://doi.org/10.3390/xxxxx

Academic Editor: Hicham Fenniri

Received: 12 June 2021
Accepted: 16 August 2021
Published: 19 August 2021

Publisher's Note: MDPI stays neutral with regard to jurisdictional claims in published maps and institutional affiliations.

Copyright: © 2021 by the authors. Submitted for possible open access publication under the terms and conditions of the Creative Commons Attribution (CC BY) license (http://creativecommons.org/licenses/by/4.0/).

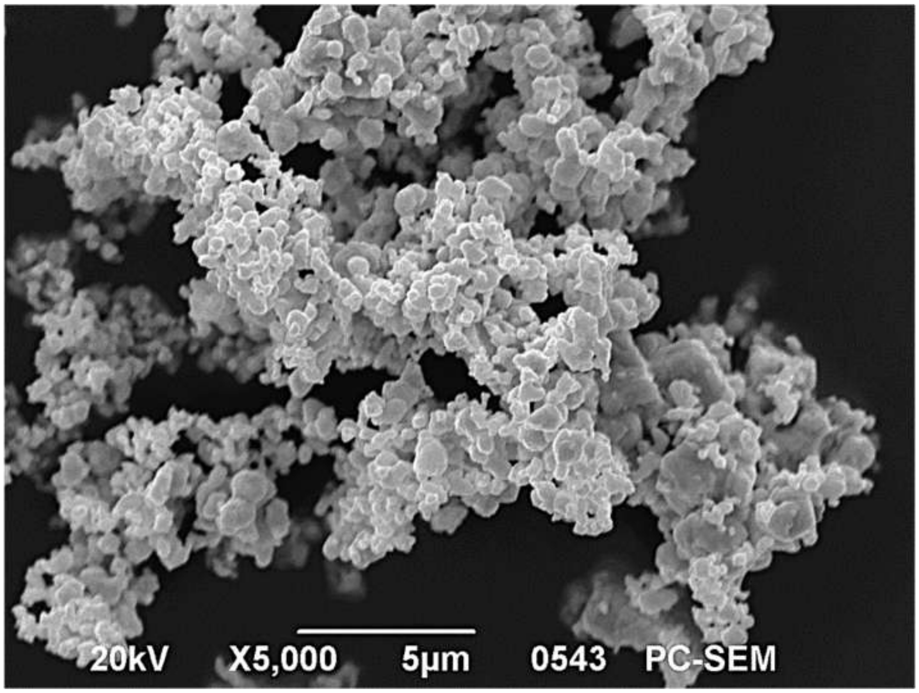

Figure S1. SEM image of WCNP.

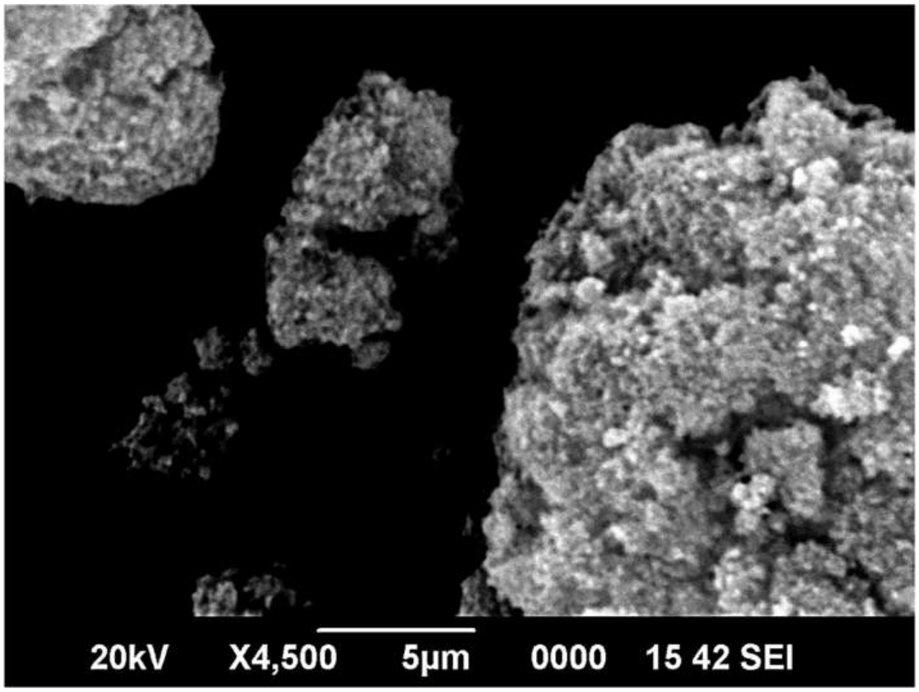

Figure S2. SEM image of AVNP2.

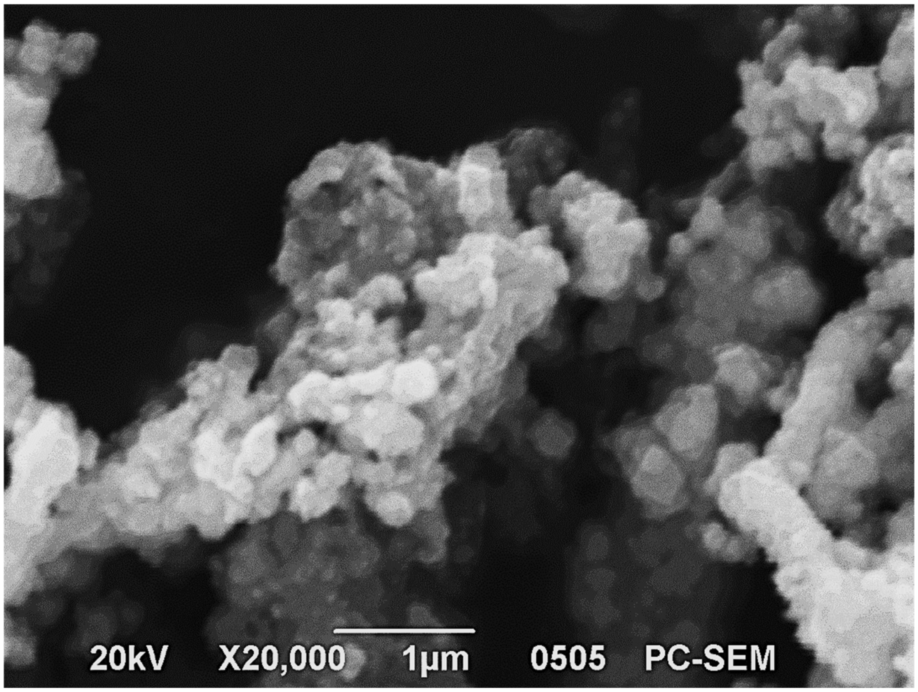

Figure S3. SEM image of CuNP.

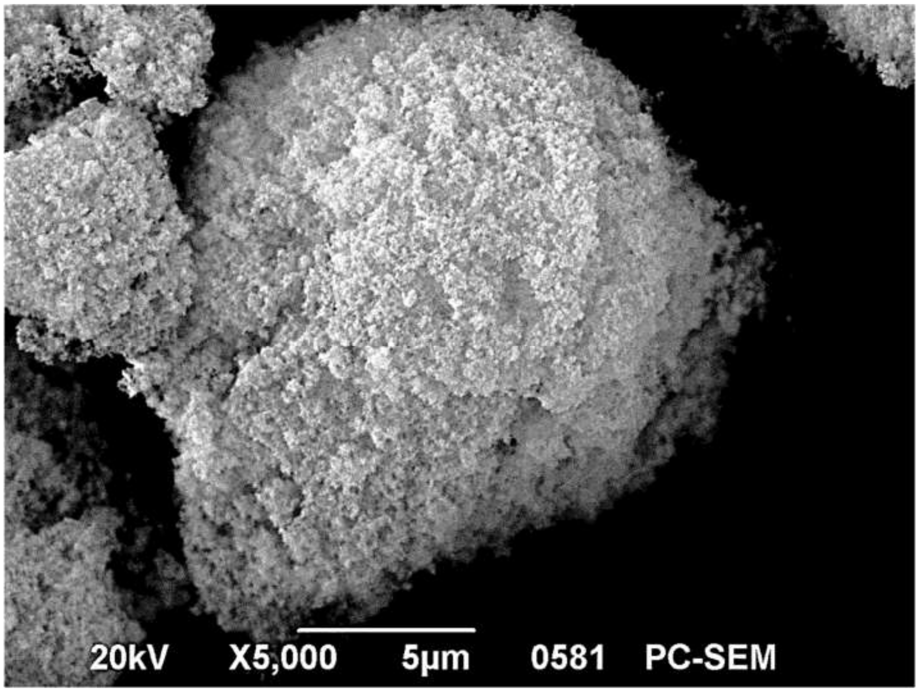

Figure S4. SEM image of CuONP.

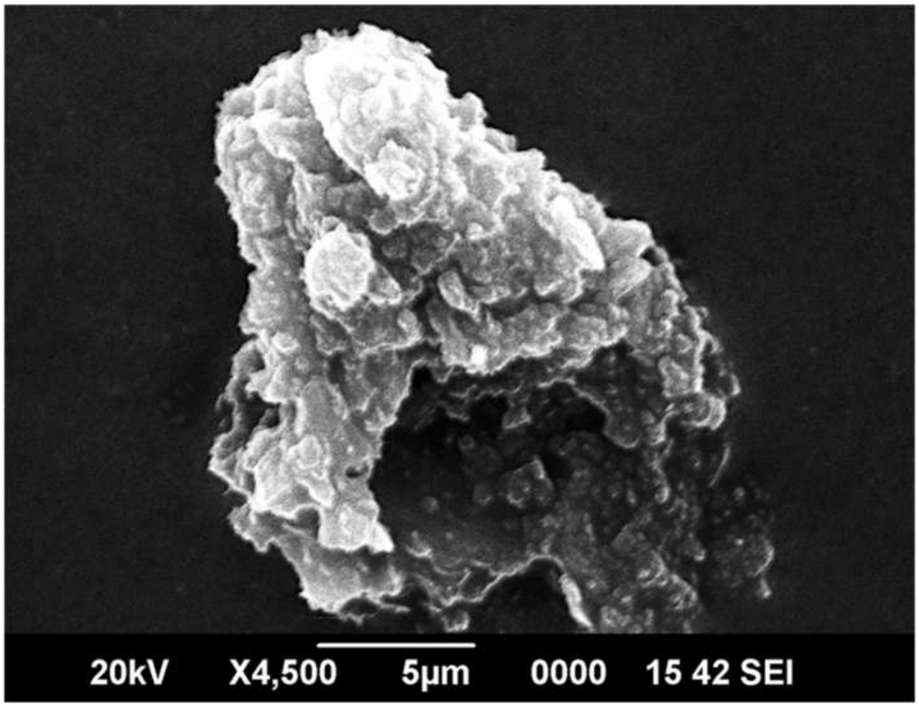

Figure S5. SEM image of ZnONP.

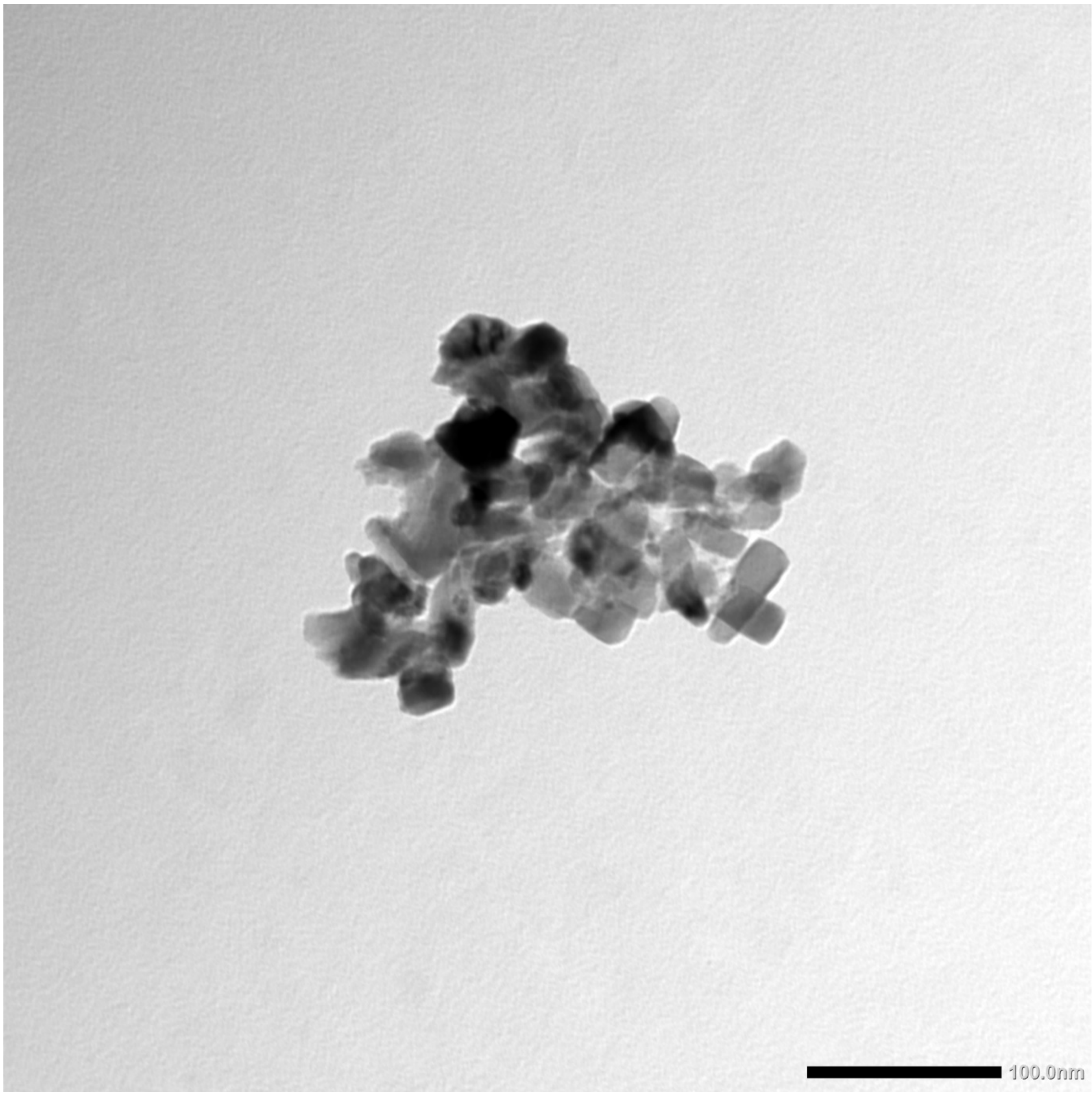

Figure S6. TEM image of CuNP (bright field).

)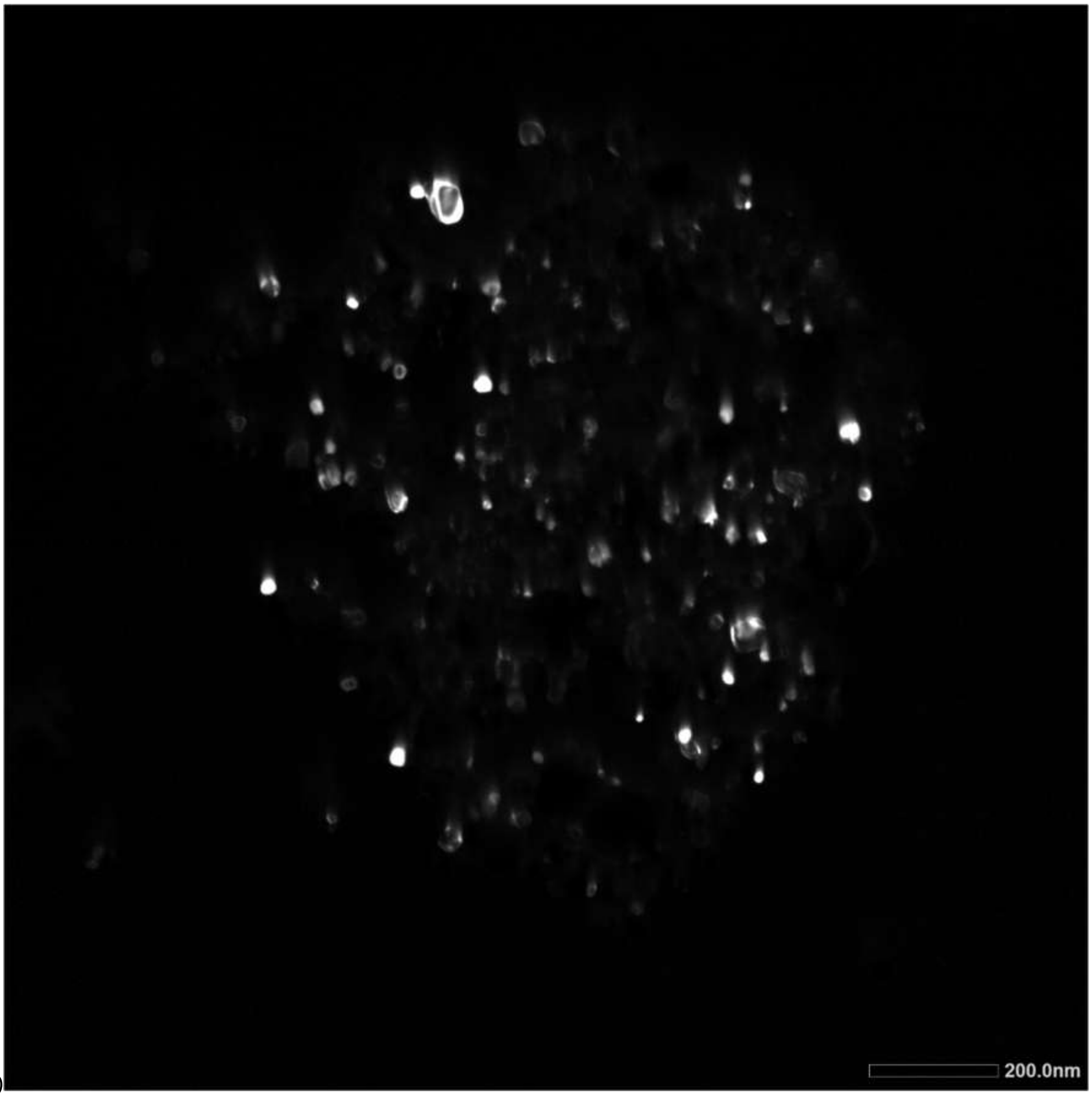

Figure S7. TEM image of CuONP (bright field).

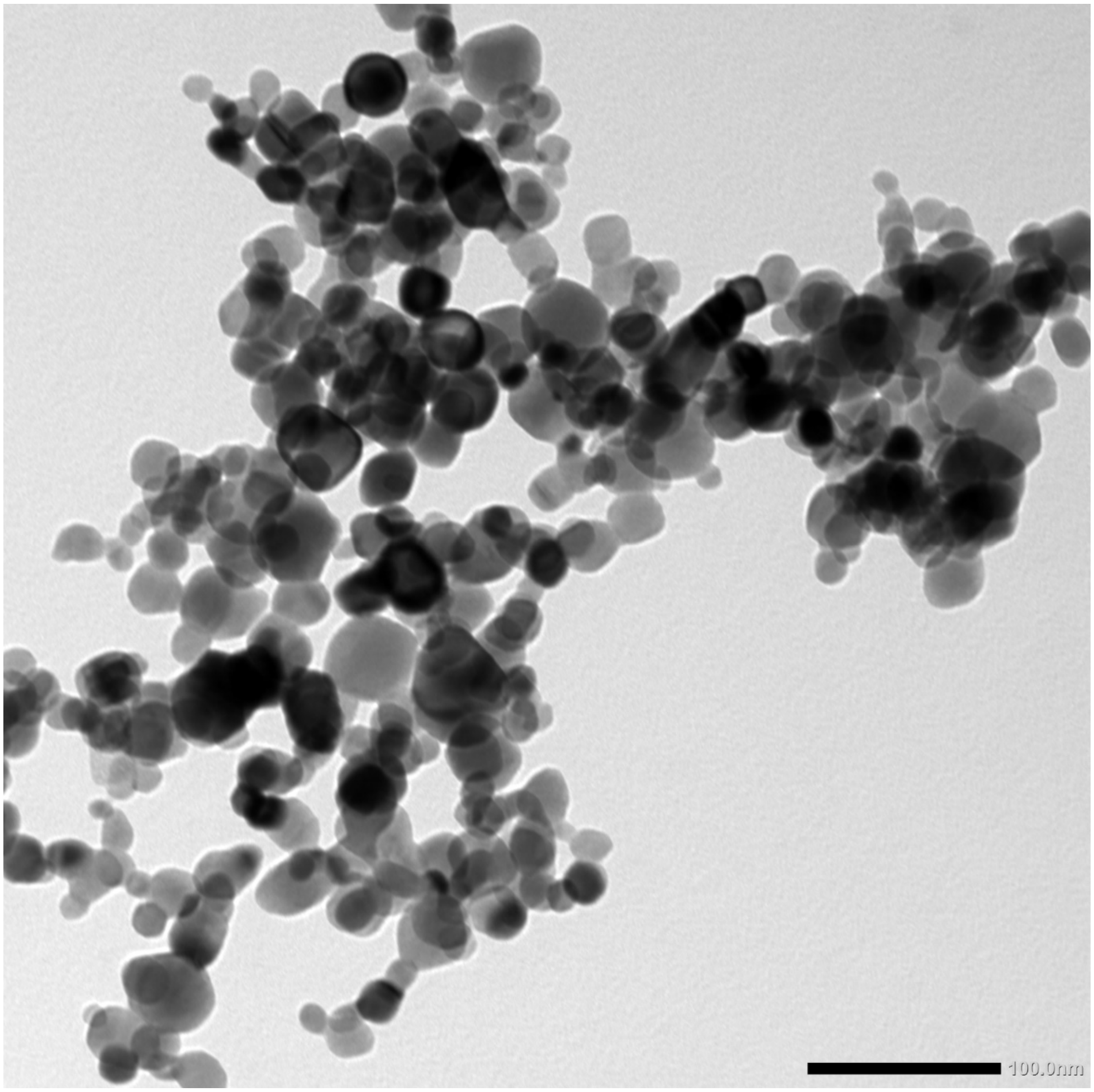

Figure S8. TEM image of CuONP (dark field).

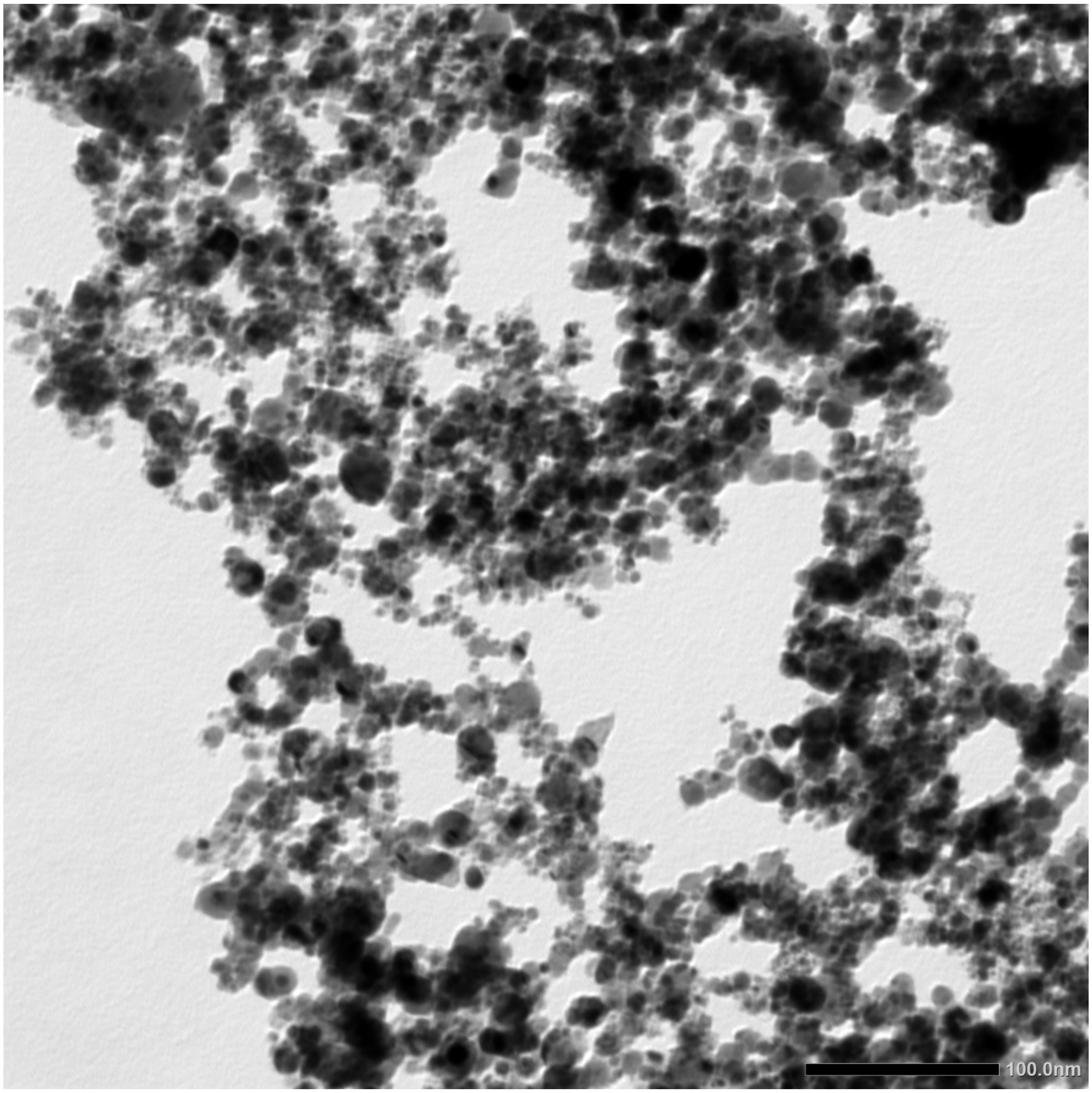

Figure S9. TEM image of AVNP2 (bright field).

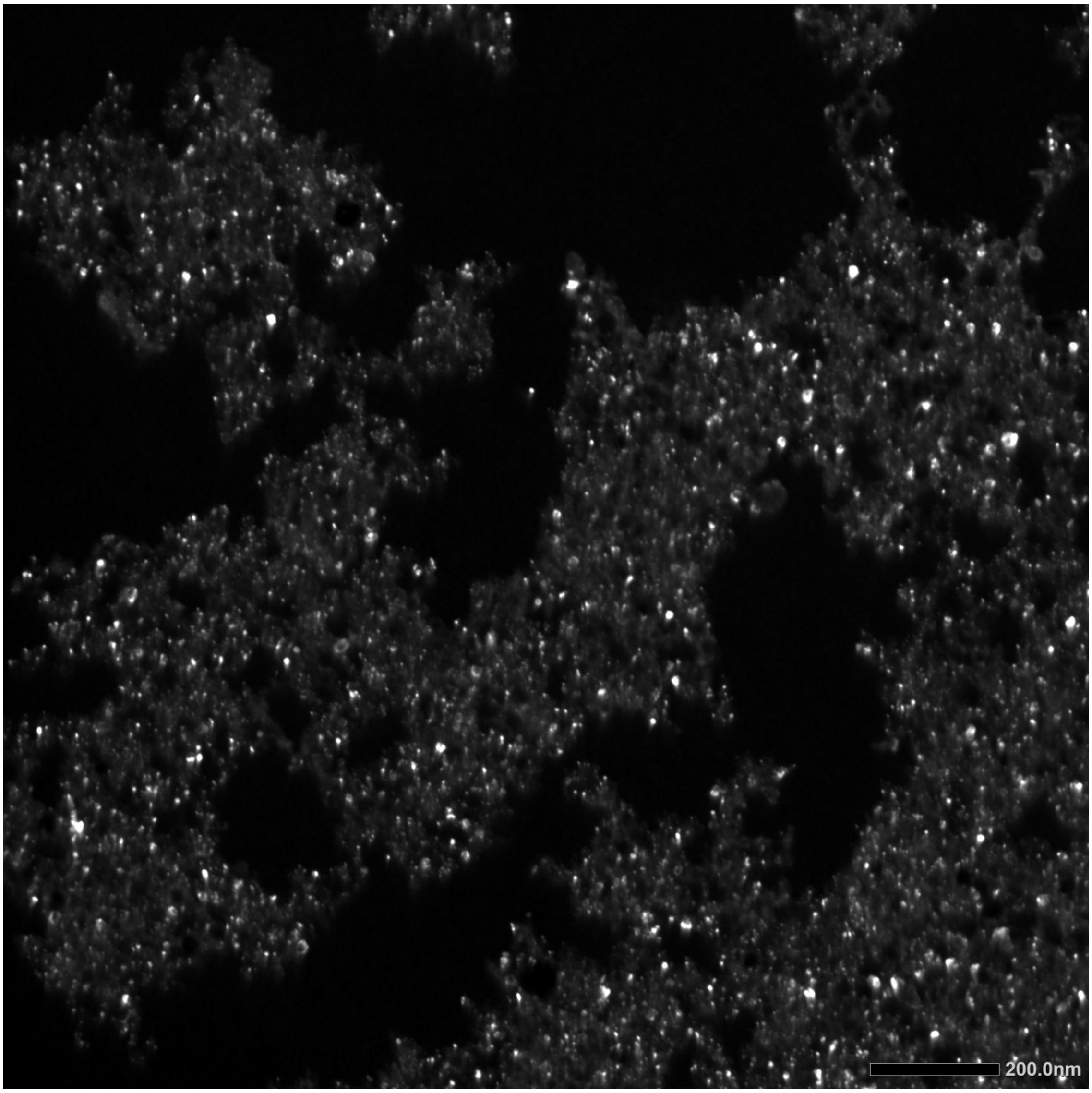

Figure S10. TEM image of AVNP2 (dark field).

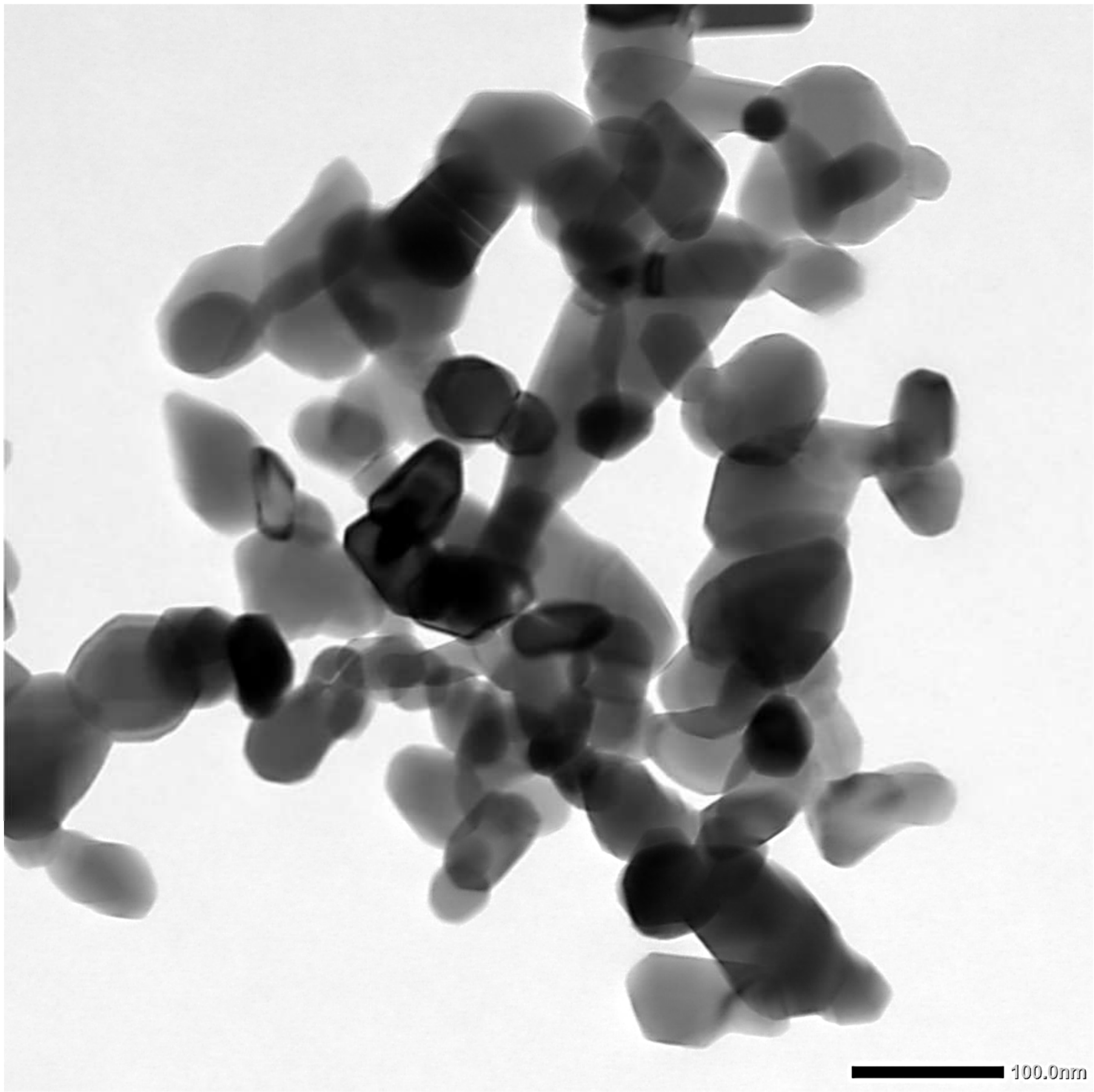

Figure S11. TEM image of ZnONP (bright field).

Fully digested Blank HNO_3

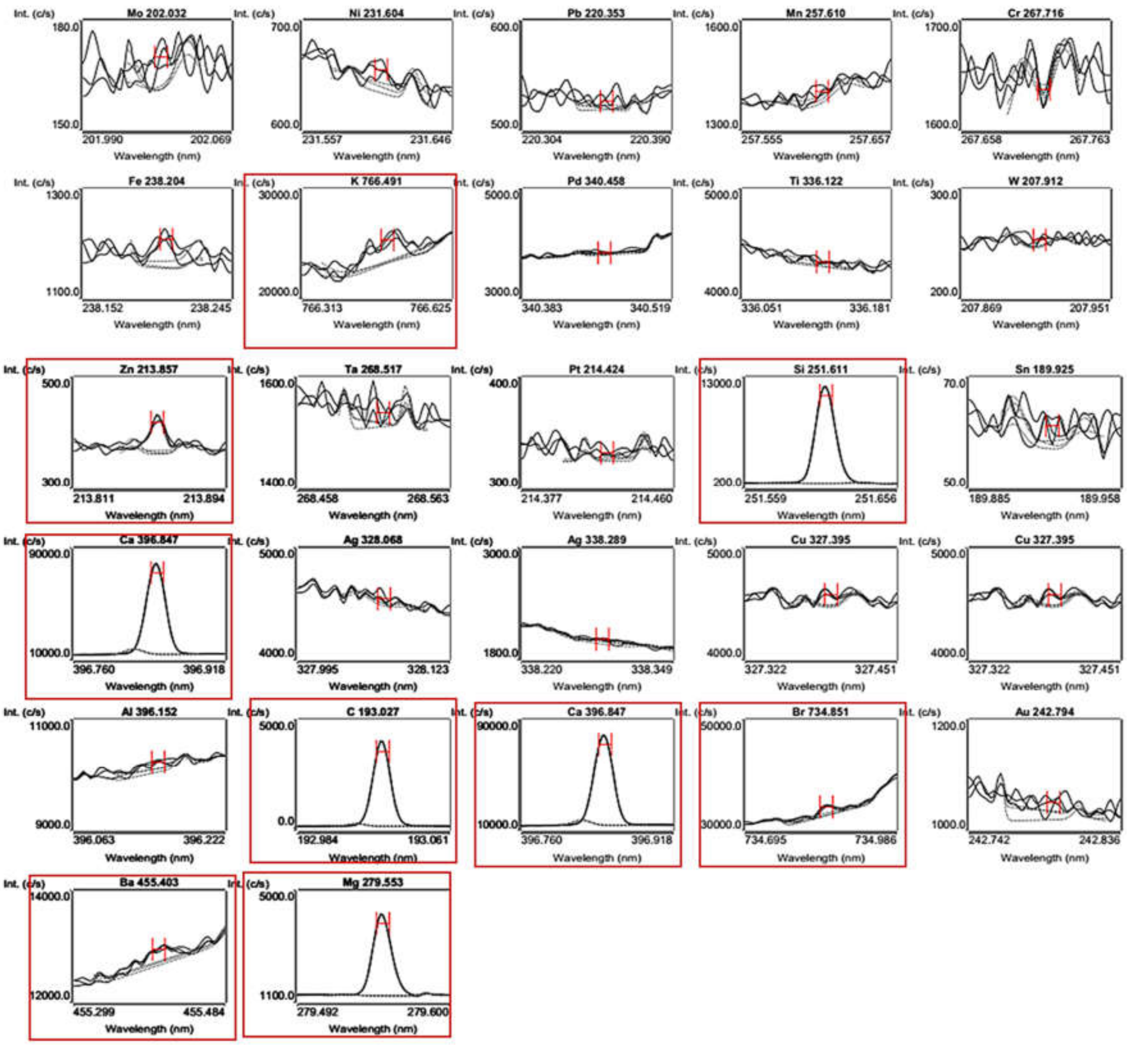

Figure S12. Elemental analysis of blank nitric acid (HNO_3) sample, where each optical emission spectrum (OES) shows the detected signal of the corresponding traced metal. As can be seen, the blank nitric acid was found to contain Zn, Si, Ca, Mg. It is worth noting that these signals also appeared in the subsequent results obtained from all AVNP samples (Figures S12, S13 and S14), as nitric acid was used for the digestion process, these observed signals therefore were regarded as blank signals.

Fully digested CuNP

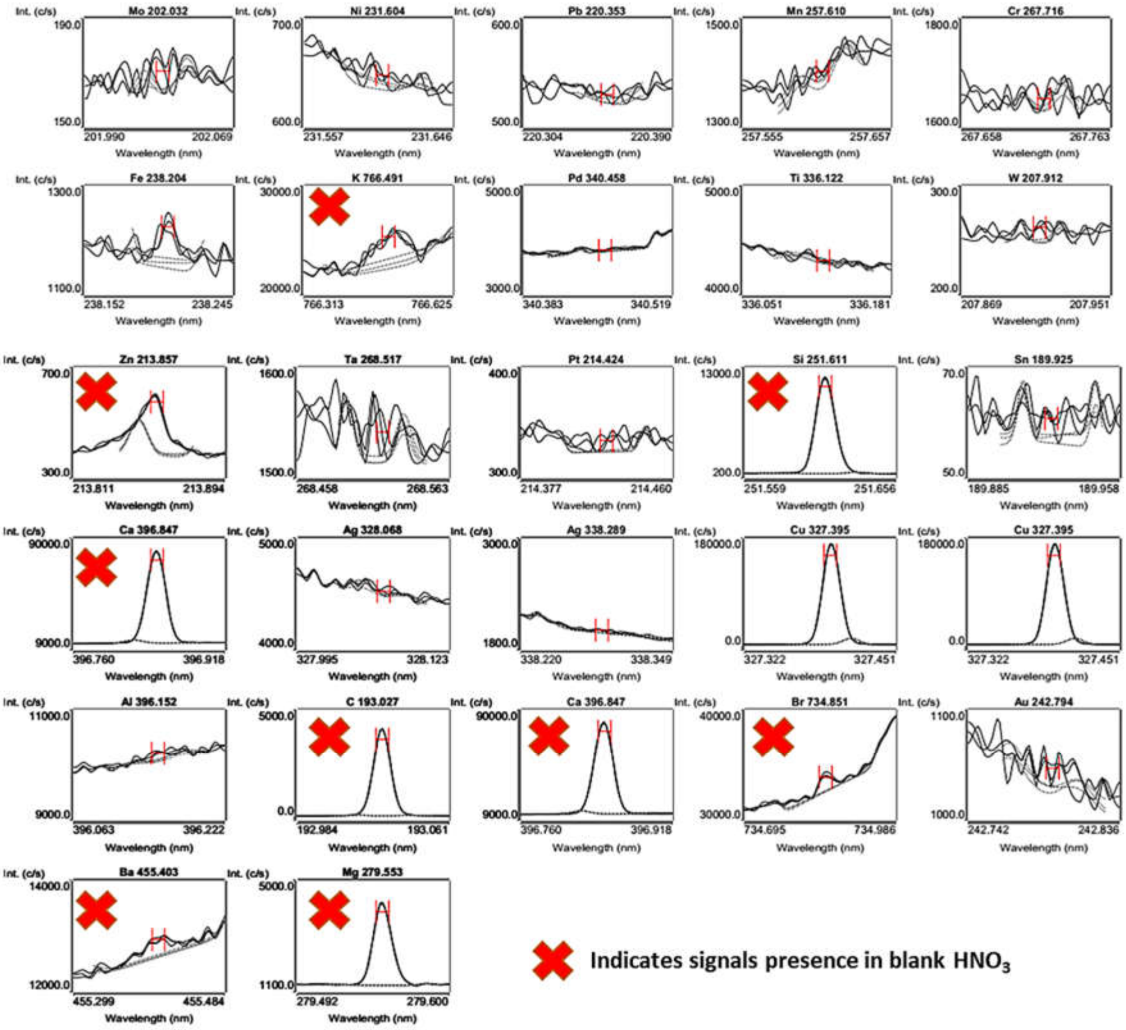

Figure S13. Metal trace elemental analysis of fully digested CuNP using ICP-OES. Apart from a trace of Iron (Fe), Copper was the only element detected in the fully digested CuNP sample.

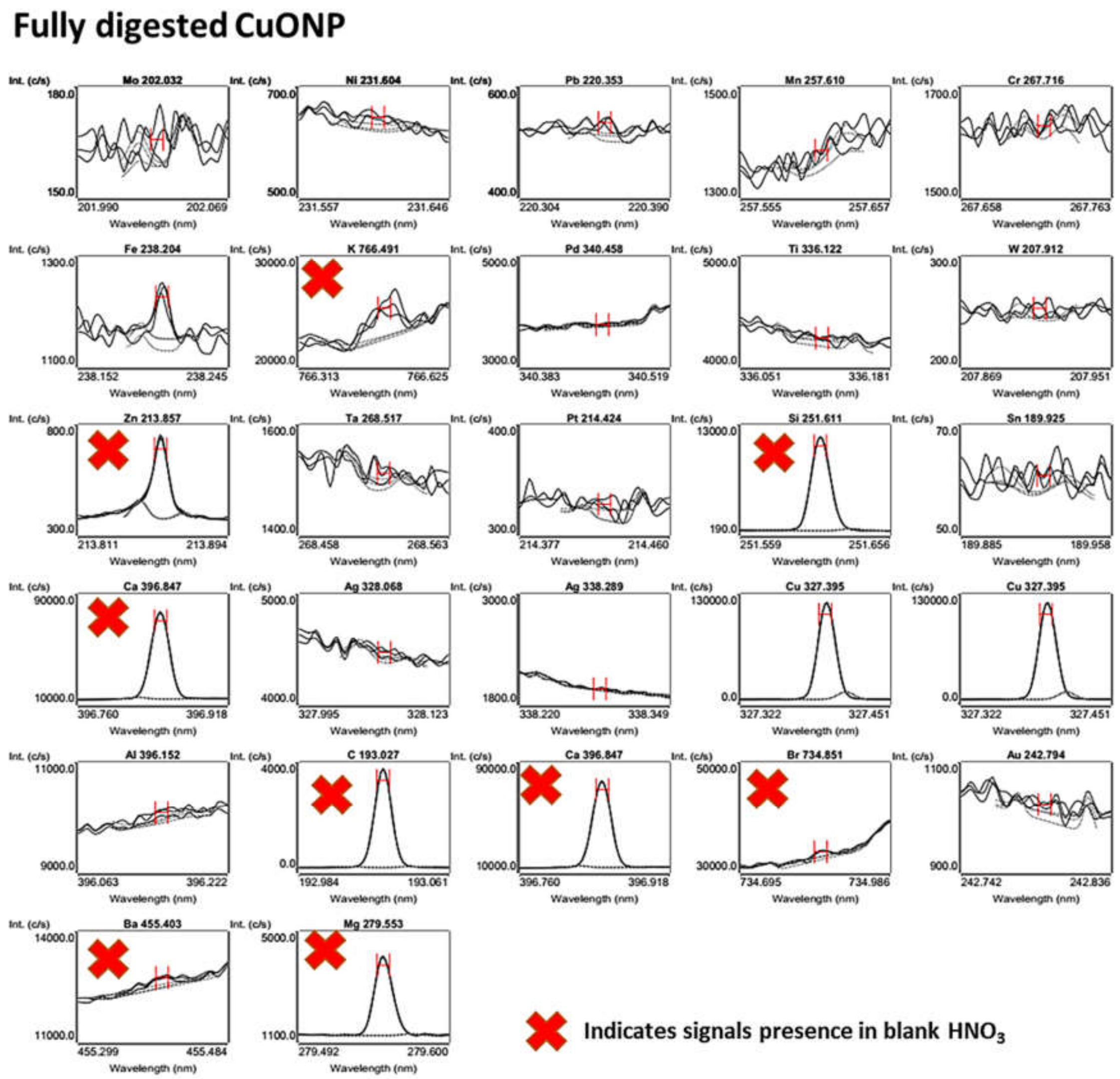

Figure S14. Metal trace elemental analysis of fully digested CuONP using ICP-OES. The manufacturing method, morphological and antimicrobial studies of CuONP have been previously published by Ren *et al.*, 2009 (DOI: 10.1016/j.ijantimicag.2008.12.004).

Fully digested AVNP2

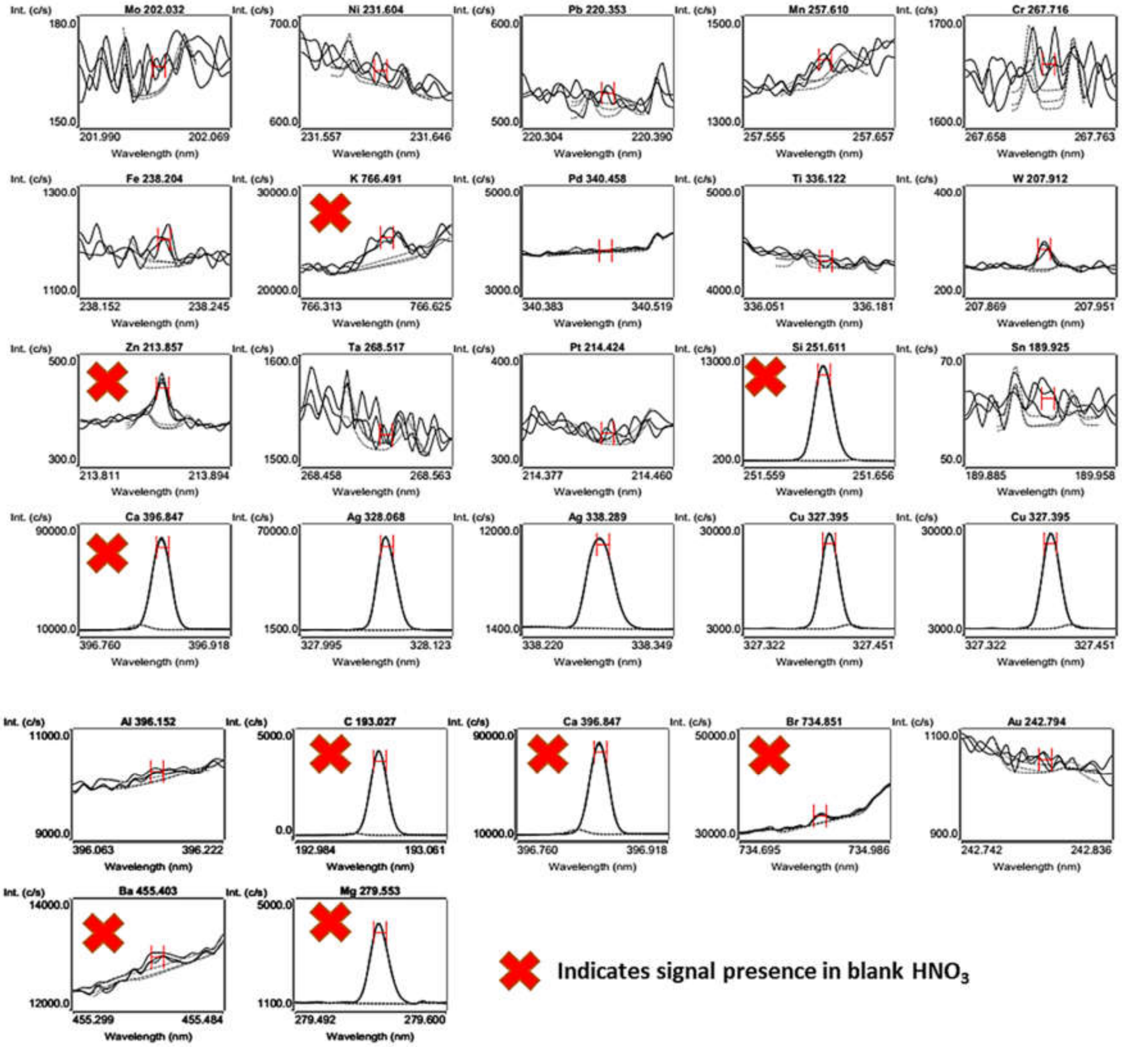

Figure S15. Metal trace elemental analysis of fully digested AVNP2 using ICP-OES. The composition of AVNP2 has been previously studied, further structural characterization was published by Cheong *et al.*, 2017 (DOI: 10.3390/nano7070152).
